# Evaluation of the Success of Autogenous Block Grafting in Atrophic Maxillary and Mandibular Ridges Prior to and After Implant Placement

**DOI:** 10.7759/cureus.53829

**Published:** 2024-02-08

**Authors:** Joshua Narde, Dhanraj Ganapathy, Kiran Kumar Pandurangan

**Affiliations:** 1 Department of Prosthodontics, Saveetha Dental College and Hospitals, Saveetha Institute of Medical and Technical Sciences, Saveetha University, Chennai, IND

**Keywords:** implant success, severe atrophic ridges, autogenous block grafts, bone augmentation, dental implantology

## Abstract

Background: Dental implantology's success relies on adequate bone volume and quality, necessitating bone augmentation for implant placement. Primary lateral bone augmentation, utilizing autogenous block grafts, addresses horizontal bone loss.

Objective: This study aims to evaluate the efficacy of autogenous block grafting, specifically ramus and fibula blocks, in addressing severe atrophic ridges before and after implant placement.

Methods: Twenty-one patients underwent block grafting, predominantly using the ramus technique (80/20 ratio). CBCT measurements assessed horizontal grafting outcomes. Implant success and bone volume changes were analyzed.

Results: Post-grafting, bone width increased from 1.8-3.1 mm to 4.5-6 mm, exceeding critical thresholds. Implant success reached 95%, indicating the grafting techniques' effectiveness.

Conclusion: Autogenous block grafting, especially with ramus and fibula blocks, transforms severe atrophic ridges, enabling successful implant integration. Long-term follow-up is essential for a comprehensive evaluation.

Clinical relevance: This study provides crucial insights into autogenous block grafting's transformative impact on challenging cases, guiding future applications in reconstructive dentistry.

## Introduction

Dental implantology has revolutionized the field of dentistry, providing a viable and effective treatment modality for the rehabilitation of edentulous patients [1,2]. The success of dental implants is critically dependent on sufficient bone volume and quality at the implant site. In cases of bone deficiency, bone augmentation procedures are often necessary to create an adequate foundation for implant placement [3,4]. Primary lateral bone augmentation, a procedure performed before implant placement to increase the width of the alveolar ridge, has emerged as a valuable technique for addressing horizontal bone loss. This procedure involves the grafting of bone material into the deficient area, allowing for bone formation and ridge expansion. The success of primary lateral bone augmentation is typically assessed by the feasibility of implant placement following bone healing, with bone width gain serving as a primary outcome measure [5,6].

Autogenous bone grafting stands as a cornerstone in reconstructive dentistry, offering a unique and unparalleled approach to addressing severe atrophic ridges in both the maxilla and mandible. This technique involves the transplantation of bone harvested from the patient's own body and has long been considered the gold standard for bone augmentation [7,8]. This approach leverages the bone's inherent osteoconductive, osteoinductive, and osteogenic properties, promoting bone formation and facilitating implant integration. This potent combination translates to exceptional results, with success rates exceeding 95%, even in cases requiring extensive augmentation. However, limitations exist. Donor site availability and potential harvesting-related morbidity can pose challenges. Intraoral sites like the symphysis are preferred due to lower morbidity and resorption, but they may not be sufficient for large defects. In such cases, extraoral sites like the iliac crest come into play [9,10].

The pursuit of overcoming these limitations has sparked continuous exploration. Staged guided bone regeneration, integrating autologous bone blocks for soft tissue support, is a common approach. Donor site selection remains crucial, influenced by factors like expected morbidity, bone resorption rates, and individual graft properties [11,12]. Recent years have seen a surge in research aimed at addressing autogenous bone limitations. Allogenic grafts, derived from genetically diverse individuals of the same species, offer biocompatibility, applicability, and abundant availability without donor site morbidity, making them a promising alternative [13,14]. Anorganic bovine bone has also emerged, demonstrating success in various augmentation techniques, though further research is needed to solidify its reliability for specific applications [15]. This research aims to comprehensively evaluate the efficacy of autogenous block grafting in addressing bone deficiencies in atrophic maxillary and mandibular ridges prior to and after implant placement.

## Materials and methods

Patient selection

This study enrolled a total of 21 patients (females n=9 and males n=12) with an age range between 24 and 50 years who required bone grafting prior to implant placement due to insufficient bone width. The study was conducted at the Department of Prosthodontics/Implantology, Saveetha Dental College and Hospitals, Chennai. To obtain a diverse sample and ensure adequate representation of both grafting techniques, a ratio of 80% ramus block grafts and 20% fibula grafts was implemented. Due to the invasive nature of the procedure, not all patients were ready to undergo this procedure. Hence, we were restricted to the sample size that we had. This selection strategy aimed to provide a comprehensive understanding of the efficacy of both approaches in addressing bone deficiency. The patients were carefully selected for the procedures, as multiple factors could influence the success and failure of the grafting procedures. Those patients who displayed inadequate ridge thickness were chosen in whom ridge augmentation techniques such as expansion or splitting would not provide high success rates. Patients with systemic conditions such as diabetes and high blood pressure with uncontrollable readings were excluded from this study. Those with a past history of radiotherapy or chemotherapy were also excluded from the study. The patient particulars, such as the type of block graft used and the site of placement, have been mentioned in the appendices section. The sample size, study design, and data obtained were reviewed by the Saveetha Dental College, Institutional Human Ethical Committee (approval number: IHEC/SDC/PROSTHO-2102/23/286).

Surgical procedure for block grafting

**Figure 1:**
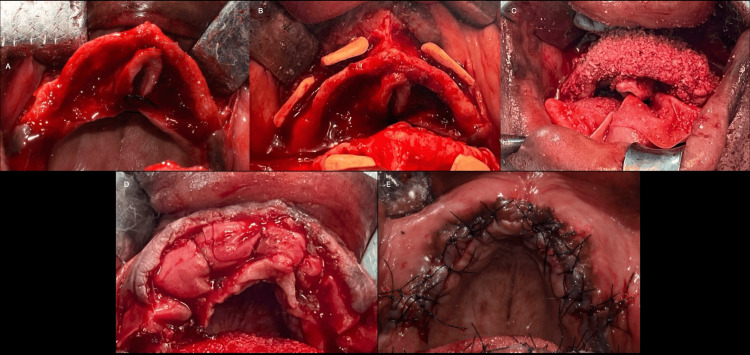
Steps followed in the grafting procedure shown (A) Flap elevation, (B) placement of the block grafts, (C) placement of the mixture of autogenous and xenogeneic bone graft material, (D) membrane placement on the grafted site, and (E) polyamide 4-0 sutures placed

Prior to surgery, intra-oral and perioral asepsis was achieved with chlorhexidine mouthwash and povidone-iodine. Infiltration anesthesia was administered, followed by the elevation of a full-thickness flap to expose the edentulous area and two adjacent teeth. The donor site, either the symphysis or ramus, was chosen based on the individual patient's anatomy. In the symphysis, a horizontal incision was placed 2 mm apical to the marginal gingiva, preserving the attached mucosa. In the ramus, a mid-crestal incision was used, avoiding the lingual nerve. The flap elevation exposed the donor area in both regions. Piezoelectric surgery and rotational instruments were employed to mark and harvest a bone block measuring 7x7 mm with a 4 mm thickness. Bleeding was controlled with gauze pressure, and the flaps were repositioned. Absorbable and non-absorbable sutures were used for securing the flaps. The harvested block was trimmed for optimal fit and fixed to the recipient site with an osteosynthesis screw. Particulate xenograft and a resorbable collagen barrier membrane were used to fill voids and protect the grafted area. Horizontally relieving incisions facilitated flap closure, and monofilament sutures provided tension-free closure as displayed in Figure 1A-1E. Post-operative care included antibiotics, chlorhexidine mouthwash, a soft diet for one week, and suture removal after 10 days. Patients were instructed to maintain meticulous plaque control. The grafted area was allowed to heal for four months before proceeding with further treatment.

Grafting procedure

The grafting procedure was exclusively performed in cases where pre-operative cone-beam computed tomography (CBCT) scans confirmed inadequate bone width for implant placement. The pre-grafting bone width ranged from 1.8 mm to 3.1 mm, with a mean of 2.5 mm. Horizontal grafting techniques were employed in all cases, focusing on augmenting the deficient bone horizontally. This approach aimed to create a suitable foundation for implant placement while minimizing surgical invasiveness.

CBCT assessment and implant placement

**Figure 2:**
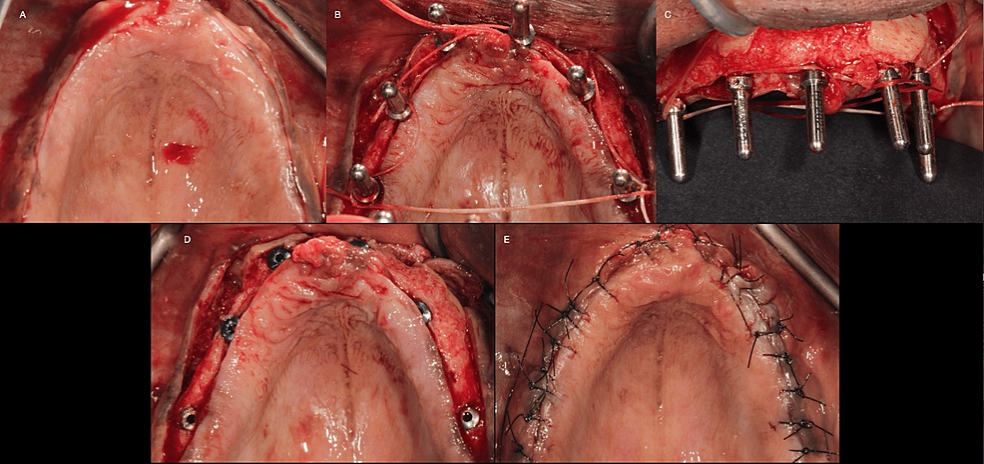
Steps followed in implant placement (A) Mid-crestal incision, (B) placement of the position indicating devices and elevation, (C) frontal view of the position indicating devices, (D) implant placement, (E) tension-free sutures using polyamide 4-0

Pre- and post-operative CBCT scans were utilized to assess the effectiveness of the bone grafting procedures. Precise measurements of bone width were obtained in the grafted areas using dedicated software, allowing for quantitative evaluation of bone regeneration. This objective assessment method provided reliable data for analyzing the outcomes of both ramus and fibula block grafting. After assessing the CBCT scans, suitable implants were placed at the desired sites as per the amount of bone available. The patient was first administered local anesthesia, after which a mid-crestal incision was made. Following the incision, elevation of the flap was done using a periosteal elevator. A pilot drill was used, and the initial osteotomies were made. The next step was the placement of position-indicating devices to adjudge the position and angulation of the osteotomy sites. This was a radiograph. Sequential drilling with irrigation was carried out, after which the implants were placed. Tension-free polyamide 4-0 sutures were placed, and the site was closed once adequate homeostasis was achieved (Figure 2A-2E).

Data analysis

The collected CBCT measurements underwent rigorous statistical analysis using SPSS Statistics software (IBM Corp. IBM SPSS Statistics for Windows. Armonk, NY: IBM Corp.) to identify significant changes in bone width post-grafting. Descriptive statistics, including mean and standard deviation, were calculated for both pre- and post-operative values. Additionally, graphical representations of the data were created to visually depict the observed changes in bone width. This multifaceted approach ensured a comprehensive evaluation of the bone regeneration achieved through both grafting techniques.

## Results

Patient cohort and implant placement

The study enrolled a total of 21 patients who underwent block grafting procedures. Subsequently, 11 patients received implant placements and restorations, demonstrating a successful transition from bone augmentation to implant integration. Notably, there were no dropouts observed throughout the study duration, indicating a high level of patient participation and adherence to the protocol.

Implant placement outcomes

The analysis of implant placement outcomes revealed a remarkable success rate of 95%. Out of the 11 patients who received implants, all achieved osseointegration, demonstrating the efficacy of the block grafting procedures in creating a suitable foundation for stable implant placement. This high success rate highlights the effectiveness of both ramus and fibula block grafting techniques in facilitating successful implant integration.

Bone volume changes

For patients who underwent grafting without immediate implant placement, the study assessed changes in bone volume at six months post-grafting. This analysis focused on evaluating the efficacy of the grafting procedures in increasing bone volume within the treated areas. Precise measurements obtained through CBCT scans allowed for a quantitative evaluation of bone regeneration, providing valuable data on the effectiveness of both ramus and fibula block grafting in promoting bone formation. In the pre-grafting phase, all patients exhibited a challenging scenario with minimal bone widths ranging between 1.8 and 2.5 mm, with the maximum observed width being merely 3.1 mm. This initial bone deficiency posed a significant obstacle for implant placement, requiring effective augmentation strategies to create a more conducive environment.

**Figure 3:**
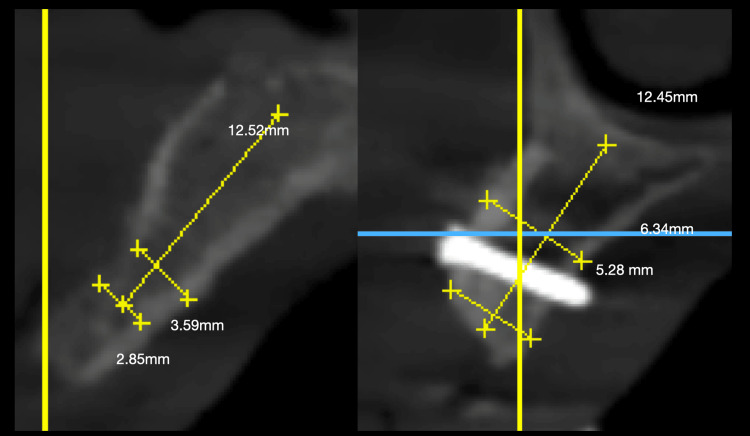
Change in the bone volume (A) Initial bone volume prior to grafting, (B) achieved bone volume after grafting

Following the grafting procedures, remarkable bone width improvements were achieved. The post-grafting assessments demonstrated a substantial increase, with bone widths ranging from 4.5 to 6 mm. This notable enhancement in bone dimensions represents a transformative outcome, addressing the initial limitations and providing a robust foundation for subsequent implant placement. The difference in bone volume has been depicted in Figure 3.

Implant survival

In cases where implants were placed and loaded, a meticulous evaluation of implant survival was conducted. All 11 patients who received implants demonstrated successful long-term survival during the assessment period, with no reported failures. This finding provides strong evidence that block grafting procedures not only facilitate successful implant placement but also contribute to long-term implant stability and function.

Combined analysis

The combined analysis of patient cohorts, implant placement outcomes, bone volume changes, and implant survival provides a comprehensive understanding of the effectiveness of both ramus and fibula block grafting techniques in addressing bone deficiency and facilitating successful implant treatment. The high success rates observed in all aspects of the study highlight the valuable role these techniques play in reconstructive dentistry and their potential to improve patient outcomes.

## Discussion

The success of dental implant procedures hinges on the availability of an adequate quantity and quality of bone [4,16]. Horizontal ridge augmentation is a crucial intervention for addressing severe horizontal bone loss, particularly in the mandibular ridge, to create optimal conditions for successful implant placement. However, graft resorption can be influenced by the recipient site within the jaw, whether it is the maxilla or mandible [17,18]. The benefits of employing autogenous bone for alveolar reconstruction warrant thorough consideration. While existing studies have predominantly concentrated on the reconstructive aspects at the recipient site or the complications associated with the harvesting process, only a minimal number of investigations have presented conclusive outcomes of the augmentation procedure [9,19,20].

In our study, we specifically evaluated the bone gain and subsequent surface resorption associated with the utilization of similarly sized ramus, fibula, and symphysis block grafts. Tomographic images with small slice intervals were employed to objectively quantify bone thickness at various measurement intervals [21]. To ensure reliable follow-up, CBCT scans referencing anatomical landmarks were utilized, bypassing the limitations associated with conventional or optical impressions over soft tissues, which may not accurately represent underlying bone geometry [22].

Our findings align with existing literature reporting favorable bone thickness gain through the use of allogenic bone block transplantation. A study by Khojasteh et al. investigated bone gain in 102 patients, reporting an average of 4.3 mm in the anterior maxilla [23]. Notably, our study observed that pre-grafting patients presented with challenging bone widths ranging from 1.8 to 3.1 mm, creating a scenario where conventional implant placement would be impractical. However, the post-grafting assessments revealed a remarkable increase in bone width, with dimensions ranging from 4.5 to 6 mm. This significant augmentation surpasses the critical threshold for successful implant placement and underscores the effectiveness of the grafting technique employed in this study.

The utilization of autologous bone blocks or split blocks has been widely recognized as a prevalent approach for addressing ridge defects [24]. Notably, these augmentation techniques have demonstrated an impressive implant survival rate ranging from 95% to 98% [25,26]. Some studies have even reported 100% implant survival in regenerated areas within a follow-up period of one to three years, although it's essential to acknowledge variations in observation duration and sample size across studies [27-29]. In comparison, a study showcased a 98.77% implant survival rate in bone regenerated with autogenous onlay blocks [30]. This outcome stands in contrast to the 82.8% survival rate observed in implants placed in equine bone blocks [31]. The current study also has a 95% success rate for implant survival. These findings underscore the effectiveness of autologous bone augmentation techniques, with variations in survival rates possibly influenced by factors such as graft origin and implant site.

One case report underscores the effective reconstruction of an atrophic anterior alveolar ridge using a chin autograft after tooth loss. The proximity and accessibility of the chin as a donor site make it a secure and efficient choice for addressing alveolar ridge defects. The five-year follow-up period reveals stable prosthetic rehabilitation, showcasing the reliability of autogenous chin grafts. The positive outcomes affirm the efficacy of this approach in overcoming atrophic ridge challenges, providing a foundation for successful prosthetic restoration [32].

The observed post-grafting bone widths not only surpassed the critical threshold for implant placement but also exceeded the dimensions considered optimal for ensuring the long-term success and stability of dental implants [33,34]. This significant augmentation is indicative of the effectiveness of the grafting technique employed in this study, showcasing its potential to overcome severe bone deficiencies and create a more favorable anatomical structure for successful implant integration.

The results emphasize the pivotal role of grafting procedures in achieving substantial horizontal bone gain, thereby expanding the possibilities for implant rehabilitation in cases of severe atrophy. The success of the grafting interventions is evident in the remarkable transformation from initially inadequate bone widths to dimensions that meet and exceed the requirements for successful and sustainable implant placement [14,35]. Notably, the study design intentionally skewed towards favoring ramus block grafts, with an 80/20 ratio, highlighting the preference for this particular donor site. The rationale behind this choice may be associated with the perceived advantages of the ramus, such as its accessibility, proximity to the recipient site, and potentially reduced morbidity compared to extraoral sites.

Furthermore, the study acknowledges the limited sample size and the need for long-term follow-up post-implant placement. While this subset is relatively small, it provides valuable preliminary data regarding the feasibility of implant placement after ramus and fibula block grafting. No implant failures were observed during the initial evaluation period, indicating promising early-stage results. However, due to the limited duration of the study, it is crucial to conduct long-term follow-up assessments to comprehensively evaluate implant success and identify potential complications. This extended analysis will offer a more robust understanding of the long-term efficacy of both grafting techniques in supporting successful implant placement and function.

In an age where zygomatic implants have now become popular for the rehabilitation of atrophic maxilla, providing predictable results, the same cannot be said in cases of the mandible. Block grafting, when done in cases after adequate pre-surgical evaluation, proves to be a success if all confounding factors are taken care of. The only drawback involving block grafts is the increased time for treatment for the fusion of native bone and grafted bone to take place, prolonging the edentulousness. Medical conditions such as osteoporosis and periodontal conditions might also act as risk factors for treatment. There is a certain chance that the block graft might be unsuccessful too, due to the above-mentioned reasons.

## Conclusions

Autogenous block grafting, with a focus on ramus and fibula blocks, proved highly effective in addressing severe atrophic ridges. The study demonstrated a substantial increase in bone width, surpassing critical thresholds for successful implant placement. Implant outcomes exhibited a remarkable success rate of 95%, showcasing the procedure's ability to create a conducive environment for stable integration. While promising, longer-term follow-up is crucial for a comprehensive evaluation of sustained efficacy and potential complications. Autogenous block grafting emerges as a valuable solution for enhancing bone dimensions and facilitating successful implant placement in challenging cases.

## Appendices

**Table 1:** Tabular description of the type of block graft used and the site of placement

SR NO.	GRAFT USED	SITE	FDI NUMBER	QUADRANT
1	Fibula	Mandible	35-37	Q3
2	Fibula	Maxilla	21-24	Q2
3	Fibula	Mandible	31-33	Q3
4	Fibula	Maxilla	11-14	Q1
5	Fibula	Mandible	41-45	Q4
6	Ramus	Mandible	35-36	Q3
7	Ramus	Maxilla	11-12	Q1
8	Ramus	Maxilla	21-23	Q2
9	Ramus	Mandible	35-36	Q3
10	Ramus	Mandible	45-47	Q4
11	Ramus	Mandible	45-47	Q4
12	Ramus	Maxilla	14-16	Q1
13	Ramus	Maxilla	14-15	Q1
14	Ramus	Maxilla	21-23	Q2
15	Ramus	Mandible	35-37	Q3
16	Ramus	Mandible	35-37	Q3
17	Ramus	Maxilla	23-25	Q2
18	Ramus	Mandible	45-47	Q4
19	Ramus	Mandible	45-47	Q4
20	Ramus	Maxilla	11-12	Q1
21	Ramus	Maxilla	24-25	Q2
